# Life Postdiagnosis: Female Adult Breast Cancer Survivors' Experience With Physical Activity—A Qualitative Systematic Review

**DOI:** 10.1155/tbj/6926093

**Published:** 2025-09-26

**Authors:** Neeve Brown, Fiona Muirhead

**Affiliations:** University of Strathclyde, Glasgow, UK

## Abstract

**Background:** With increasing survival rates of breast cancer, there is a need for more research to understand the experiences of survivors. Previous quantitative studies have shown that physical activity can be beneficial for breast cancer survivors. However, a qualitative perspective is essential to create appropriate adaptations for this population. This study aims to develop a deeper understanding of the experiences of female adult breast cancer survivors with physical activity in their postdiagnosis lifestyle.

**Methods:** This study followed a qualitative systematic review methodology. In January 2024, six databases (APA PsycInfo, CINAHL Plus, OVID Medline, Scopus, SPORTDiscus and Sports Medicine an Education Index) were searched using aim-specific key terms. Ten studies, comprising a total sample of 200 participants, met the inclusion criteria. Quality appraisal, data extraction and synthesis stages were conducted.

**Results:** Five main themes emerged during the synthesis stage: (1) Outcomes of Physical Activity Participation, (2) Barriers to Physical Activity, (3) Postdiagnosis Balancing Act, (4) Needs for Future Physical Activity Programs and (5) Next Steps for Breast Cancer Survivors. Additionally, 15 subthemes were identified.

**Conclusion:** Overall, breast cancer survivors reported positive experiences with physical activity, leading to a desire to maintain an active lifestyle. However, barriers such as treatment side effects, unmet needs for advice from health services and challenges in daily life postdiagnosis were identified. Future research should explore the implementation of specific national guidelines and recommendations for survivors postdiagnosis to overcome these barriers and enhance the quality of survivorship care.

## 1. Introduction

Breast cancer (BC) is the most prevalent form of cancer globally [1]. By 2045, BC incidence is projected to increase by 40%, resulting in approximately 3 million new diagnoses annually [2]. BC's clinical presentation, treatment outcomes and prognosis are shaped by a combination of genetic, lifestyle, environmental and demographic influences [3, 4]. Key modifiable risk factors—including obesity, smoking, diabetes and HIV infection—have been consistently linked to increased BC mortality [5]. Obesity is also correlated with higher BC incidence in developed countries [6, 7]. Despite the increase in diagnoses, BC survival rates have also risen. In the UK, survival rates have nearly doubled from 40% in the 1970s to 78% in the 2010s for 10 years post-diagnosis [8]. This improvement is due to advancements in medical technology, early detection, accessible treatments, preventative procedures and enhanced research [1].

Recent evidence indicates that breast cancer survivors (BCS) can experience long-lasting side effects from treatments like chemotherapy, including fatigue, lymphedema, infertility, chronic pain, early menopause, sexual dysfunction, body image issues and accelerated aging [9–11]. Additionally, BCS may face general survivorship struggles such as trauma, reduced quality of life, altered self-perception and social and work difficulties [11]. Therefore, high-quality, accessible and specific survivorship care is essential to support BCS post-diagnosis and post-treatment.

Promoting positive lifestyle choices, including physical activity (PA), nutrition, social connection, adequate sleep, stress management and avoidance of toxic substances, can improve survival and quality of life for BC patients [12].

A recent systematic review (SR) of moderate-intensity exercise interventions in BC patients and survivors showed improvements in cardiorespiratory fitness, strength, fatigue and health-related quality of life [13]. Higher levels of PA are associated with lower risks of BC and all-cause mortality [14]. However, only 8% of BCS meet national PA guidelines [15]. The ASCO now recommends exercise during treatment to improve quality of life, reduce treatment toxicity and control cancer progression [16].

Although many BC patients report engaging in at least one positive dietary or PA behaviour following diagnosis or treatment, recent findings reveal that several treatment-related challenges—such as fatigue, stress, altered appetite and taste, pain and discomfort—often hinder the consistent adoption of these healthy lifestyle changes [17]. Despite the known benefits of PA [13, 14], research has shown that individuals undergoing treatment for BC often experience a decline in engagement with PA and other healthy lifestyle behaviours. Moreover, these reduced levels of participation frequently persist even after treatment has concluded [18].

While quantitative findings provide clarity, qualitative data from the lived experiences of BCS offer valuable insights into the low uptake of PA post-diagnosis, understanding these experiences can inform tailored interventions to enhance survivorship care. While a recent review by Lavallée et al. [19] synthesised the barriers and facilitators to engaging in and participating in PA during treatment, understanding the barriers and facilitators to PA among BCS post-treatment is crucial for several reasons. While the benefits of PA during and after cancer treatment are well documented [12], the transition from active treatment to survivorship for BCS may introduce new or intensified barriers to PA. For example, the facilitators that encourage PA during treatment, such as structured exercise programs and healthcare provider support [20] may not be as readily available or effective in the post-treatment phase. Moreover, qualitative insights from BCS can provide a deeper understanding of their lived experiences, motivations and preferences regarding PA. These insights will be invaluable for designing patient-centred interventions that resonate with BCS and address their specific needs and concerns.

Therefore, an updated qualitative SR is needed to bridge the gap between quantitative findings and BCS' post-diagnosis PA experiences. The aim of this qualitative SR is to develop a deeper understanding of the experiences of female adult BCS with PA in their post-diagnosis lifestyle.

## 2. Methods

### 2.1. Study Design

This study follows the 24-step SR methodology outlined by Muka et al. [21]. The Preferred Reporting Items for Systematic Reviews and Meta-Analyses (PRISMA) 2020 statement was used as a guide and checklist to ensure a clear and accepted structure [22]. The guide by Butler et al. [23] was adapted to align with the qualitative design. The Sample, Phenomenon of Interest, Design, Evaluation, and Research type (SPIDER) framework was used to formulate the aim (see Table 1), as it is more suitable for qualitative data than the Population, Intervention, Comparison, Outcome (PICO) tool [24]. The PRISMA flow diagram was employed to provide a clear summary of the search strategy process and results for replicability [22].

### 2.2. Search Strategy

This review utilised six electronic databases for the search strategy: American Psychological Association (APA) PsycInfo, Cumulative Index to Nursing and Allied Health Literature (CINAHL) Plus, OVID Medline, Scopus, SPORTDiscus and Sports Medicine & Education Index. These databases were selected for their relevance to the review's aims and the SPIDER framework. Consistent key terms were used across all databases to maintain standardised practice (see Table 2). Proximity search techniques, recommended by an experienced Research Librarian at the University of Strathclyde, included searching for ‘breast cancer' and ‘surviv^∗^' within three words of each other, with conjunction terms adapted for each database (see Table 2). Additional key terms included experienc^∗^ AND ‘physical activity' OR ‘PA' OR sport^∗^ OR fitness OR exercis^∗^. Filters applied in each database reflected the study's aims and inclusion/exclusion criteria: published in the last 10 years (January 2014–January 2024), English language and peer-reviewed (where applicable). The search strategy was conducted independently by the lead author (NB) on January 30, 2024.

### 2.3. Eligibility Criteria and Selection

**Figure 1:**
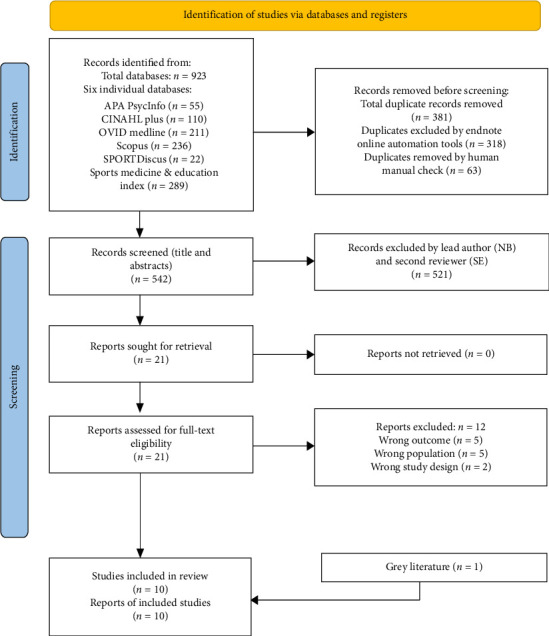
PRISMA flow diagram of screening and selection processes.

Once the searches were conducted and key papers saved to Endnote, the initial hits were exported from each database and imported into separate folders. These were then combined into one overall folder (*n* = 923), and duplicates were removed using an automated tool (318 removed). A manual check ensured all duplicates were excluded, leading to the removal of an additional 63 articles. The remaining 542 articles were exported into Rayyan software for screening. The lead author (NB) independently screened all papers based on titles and abstracts, following the identified inclusion and exclusion criteria (see Table 3). A second reviewer (SE) independently screened 20% of the papers to increase reliability and reduce bias, resulting in a 100% agreement rate. From this screening stage, 521 articles were excluded for various reasons, including wrong population, wrong outcome, or wrong study design. The remaining 21 articles underwent full-text reviews by the author (NB) to assess eligibility, leading to nine articles meeting the inclusion criteria. One additional article was discovered separately and included as grey literature, resulting in a final selection of 10 articles. The PRISMA flow diagram summarises the screening and selection stages during the search strategy process (see Figure 1).

### 2.4. Quality Appraisal

The final articles were appraised using the Critical Appraisal Skills Programme (CASP) checklist, supported by the Cochrane Qualitative and Implementation Methods group [25]. The modified version by Long et al. [25] includes an additional question and a fourth response option, ‘somewhat', indicating a reasonable attempt at fulfilling a quality domain with clear strengths and limitations. The assessment involved scoring 11 questions regarding methodological quality as ‘yes', ‘can't tell', ‘no' and ‘somewhat'. These questions addressed research design, recruitment strategy, data collection, ethics, data analysis, and presentation and interpretation of findings. The modified CASP checklist template used in this study is available in the supporting information (available here).

Each domain includes ‘hints' for further clarification, encouraging appropriate assessment. The quality of the articles was determined by prioritising the rigor of data analysis, which affects the reliability and credibility of conclusions. A second reviewer (FM), experienced in qualitative data handling and a Senior Lecturer at the University of Strathclyde, collaborated with the author (NB) to assess the quality of all papers.

### 2.5. Data Extraction and Synthesis

Data was extracted by examining the abstracts, methods and results of each article independently by the author (NB). The data was summarised using an adapted Cochrane Handbook template, which recommended well-used data extraction domains specific to qualitative studies and was tailored to this review's aim [26]. Following Butler et al. [23], the author applied data synthesis methods similar to a meta-summary, analysing content to create an aggregation of the overall findings. The synthesis followed adapted Thomas and Harden [27] methods, including listing all extracted main findings, grouping similar ideas and renaming the grouped findings to achieve suitable overall representation of all collated themes. The original articles were referred to during the synthesis procedure to fully grasp and understand the resultant themes [26]. Ethical procedures were adhered to when extracting participant data in accordance with the Enhancing Transparency in Reporting the Synthesis of Qualitative Research (ENTREQ) guidelines, ensuring participant confidentiality was maintained [28].

## 3. Results

### 3.1. Study Characteristics

The 10 final studies included were: Balneaves et al. [29]; Chan et al. [30]; Hirschey et al. [31]; Inam et al. [32]; Kim et al. [33]; Milosevic et al. [34]; Owusu et al. [35]; Robinson et al. [36]; Sebri et al. [37] and Stalsberg et al. [38]. These studies were summarised in a data extraction table highlighting each study's characteristics (see Appendix A). In total, 200 BCS participated across the 10 studies, which were conducted in the USA (*n* = 3), Canada (*n* = 2), Australia (*n* = 1), China (*n* = 1), Italy (*n* = 1), Korea (*n* = 1) and Norway (*n* = 1). All articles received ethical approval. The final studies included qualitative (*n* = 8) and mixed-method (*n* = 2) designs. Data collection methods consisted of semi-structured interviews, one-to-one interviews, focus groups and qualitative questionnaires. Half of the articles (*n* = 5) were intervention-based PA experiences, while the other half (*n* = 5) focused on general PA experiences. Convenience (*n* = 4) and purposive sampling (*n* = 6) were used to recruit participants. Data analysis approaches included Thematic Analysis (*n* = 5), Content Analysis (*n* = 4) and Mixed-Parallel Analysis (*n* = 1) [38]. Inam et al. [32] included healthcare professionals alongside BCS, with data presented separately. Kim et al. [33] included four participants undergoing treatment at the time of the study. All other articles included BCS post-treatment.

### 3.2. Quality Appraisal

The quality of the final 10 articles was assessed using the CASP checklist (see Table 4), including guidance from Long et al. [25]. The assessment resulted in high- (*n* = 3), medium- (*n* = 5) and low (*n* = 2)-quality conclusions. The authors (NB and FM) appraised 100% (*n* = 10) of the included articles, achieving a high degree of agreement (90%, *n* = 9). Discrepancies were resolved through discussion. The ‘can't tell' option was used when studies lacked sufficient information. In response to Question 11 (‘How valuable is the research?'), three higher-quality articles [32, 33, 35] were deemed more valuable due to their reliability of findings. Additionally, articles without specific interventions [30, 34] were considered valuable for their generalisation of everyday PA experiences for all BCS. All final studies were included regardless of their quality conclusions.

### 3.3. Main Findings

Thomas and Harden's [27] data synthesis methods were employed to construct 5 main themes and 15 sub-themes, representing BCS' experiences with PA in the final 10 articles (see Table 5). The main themes identified were ‘Outcomes of PA Participation', ‘Barriers Surrounding PA', ‘The Post-Diagnosis Balancing Act', ‘Needs for Future PA Programmes' and ‘Next Steps for BCS'. Each theme description is presented below.

#### 3.3.1. Theme 1: Outcomes of PA Participation

The impact of group PA was reported by participants in all 10 articles. Social connections fostered a safe environment where relatable life experiences could be shared, understood and supported. Physically, PA made BCS feel stronger and increased their energy levels. Psychologically, PA improved stress management, self-efficacy and confidence.

*‘I was like a shell and now it feels like I'm filling up like I used to be. It's more like I'm becoming who I am, not this sort of shell of being weak and sick'.* [29]

Hence, both physical and psychological benefits aided BCS in reducing side-effects of treatments. Thirdly, participation also facilitated a more positive outlook on life and one's future, dwelling less on the likelihood of BC recurrence. Lastly, BSC reported positively on the ability to self-manage their participation and took comfort in their ability to sustain and take care of themselves. This self-management allowed them to be in control and was in stark contrast to their cancer diagnosis and journey, which was mostly out of their control:

*‘You really need to focus on mental benefits of exercise for breast cancer and control is a big issue too, you are dealing with not being in control, exercise is something you can do'.* [31]

#### 3.3.2. Theme 2: Barriers Surrounding PA

Six of the reviewed articles highlighted barriers to PA experienced by BCS in terms of knowledge, capabilities and commitments [29–31, 33–35]. Firstly, BCS reported receiving insufficient or incorrect information from medical staff about exercising as a survivor. For instance, Kim et al. [33] noted that one individual was advised not to use their lymphedema-affected limbs during exercise. Additionally, the opinions of family and friends, often based on misinformation, significantly impacted BCS decisions, leading some to avoid PA due to fears of potential negative impacts.

*‘I had a friend who had recurrence and she believes it was because she exercised too aggressively'.* [33]

Secondly, side effects from treatments were a significant barrier due to their substantial impact on an individual's capacity to engage in PA:

*‘I've tried exercising (for muscle strength) but my back hurts, my legs shake, and I can't really muster much strength. No vigor, and now even my knees hurt (so it's hard to do it)'.* [33]

Many noted their fatigue, pain and decreased energy resulted in a lack of motivation, significantly impacting their ability to participate in PA. Their daily lives were greatly affected by these symptoms. Additionally, the challenge of balancing family, work and social commitments was another barrier, as many individuals felt they lacked the time and energy to manage everything.

#### 3.3.3. Theme 3: Postdiagnosis Balancing Act

In half of the articles (*n* = 5), survivors mentioned the negative impact of comparing two differing situations and the need to find balance here [32–34, 37, 38]. Firstly, BCS compared their prediagnosis level of PA and abilities, with their post-diagnosis levels. This led to many individuals holding a predominantly negative view of their fitness levels postdiagnosis. Secondly, individuals compared themselves to others around them in exercise settings. This comparison resulted in individuals feeling deflated and demotivated: ‘*I feel smaller than them. You know, physically and emotionally small'.* [33]*; ‘I can't lose weight the same way as my girlfriends. Like my girlfriends have done like different programs that I haven't been able to do because I wasn't allowed to do intense cardio … It's really hard to [exercise] with somebody who isn't going through the same things or that understands what you're going through and how your body's feeling'.* [34].

Lastly, several BCS discussed conflicting priorities in life post-diagnosis. Survivors commented that post-treatment they ought to enjoy themselves, do as they please and live without restrictive PA routines:

*‘I don't want to be like ‘No I actually can't eat that because of … restrictions that I put on myself because I'm fearful that my cancer's going to come back.' And it's sort of hard that way'.* [34]

Many held the view that a healthy lifestyle equated to a high volume of PA and strict dieting, this contrasted with their desire to relax post-treatment.

#### 3.3.4. Theme 4: Needs for Future PA Programmes

Half of the articles (*n* = 5) described key elements for future programmes and interventions in outlining the needs for all BCS to positively experience PA [29, 30, 32, 33, 35]. Firstly, BCS highlighted the importance of flexibility within programmes, so it is adaptable to the varying interests, PA levels and goals of participants. Furthermore, enjoyment must be had in connection with this specificity as this would help keep participants engaged and motivated, increasing the likelihood of recurring attendance: ‘*Exercise programs should be fun and enjoyable'.* [33]. Secondly, the PA environment was found to be important to BCS. Individuals reported that participating in PA out with hospital settings and be outdoors where possible, was favourable. Lastly, the structure of sessions must be somewhat structured with professionals on hand for advice when needed:

*‘What I need 12 months on is to be able to go back and say, see this is what I'm doing, this is how I'm doing it. These are my current physical kind of limitations or pain and stiffness concerns. Can you let me know do I need to increase weights, reduce weight, change some of my exercises and that could just be once every three months'.* [32].

This PA advice must be accurate and maintain a standardised approach throughout the medical system and exercise contacts; thus, every individual has access to accurate information.

#### 3.3.5. Theme 5: Next Steps for BCS

Five articles discussed what was next for BCS in terms of involving PA in their future [29, 31, 36–38]. Multiple positive responses denoted that individuals wished to continue PA and make it part of their normal routine. A few groups of BCS also discussed continuing to exercise as a group out with the programme or intervention they were involved in: *‘We have enough participants to do something like an alumni'.* [36]*; ‘We need to support each other … we can't let everybody go on their way'.* [29]. Many survivors made aware that they wish to be included in studies where possible, to support research, findings and help other BCS too:

*‘I believe in the importance of these studies and I hope that my little contribution will be useful for me and for women who lived similar illnesses'.* [37]

## 4. Discussion

This qualitative study systematically reviewed 10 studies to develop an increased understanding of female adult BCS' experiences with PA in their post-diagnosis lifestyle. By gaining insights directly from BCS through qualitative data, the study accurately represents the experiences of this growing population. The main themes identified were: (1) Outcomes of PA Participation, (2) Barriers Surrounding PA, (3) Post-Diagnosis Balancing Act, (4) Needs for Future PA Programs and (5) Next Steps for BCS.

### 4.1. Results and Implications

The study found that PA participation positively impacts BCS, with tailored interventions helping individuals cope with exercise and improve activity levels. Some treatment side effects decreased after PA participation, reflecting existing literature on reduced stress, stronger mentality and increased energy levels [39]. PA also provided survivors with a sense of control over their post-diagnosis life, promoting independence and self-management [30–33, 36, 37]. This self-management approach aligns with Soldato et al. [11] and could reduce the burden on healthcare systems like the National Health Service (NHS) by making PA a more self-reliant element of survivorship care.

Additionally, some BCS received little to no advice or misinformation from healthcare staff regarding PA, treatment side effects and post-treatment lifestyle [30, 31, 33]. This may be due to healthcare systems struggling to keep up with the rising incidence of BC and the need to distribute accurate information to this growing population. It is crucial for healthcare services to provide precise and effective support, especially during the post-treatment stage of the cancer journey.

Overall, the findings from this qualitative SR provide valuable insights into the barriers and facilitators to PA among BCS post-treatment. This review adds to the existing body of research by highlighting the unique challenges and enablers faced by BCS in the post-treatment phase, which differ from those experienced during active treatment. One of the key contributions of this review is the identification of new or intensified barriers to PA that emerge during the transition from active treatment to survivorship. These barriers include lingering treatment side effects, fear of recurrence and changes in social support networks. This finding aligns with previous research that has documented the physical and psychological adjustments that BCS undergo during this critical period [12].

Additionally, this review highlights the need for tailored interventions that consider the facilitators to PA in the post-treatment context. While structured exercise programs and healthcare provider support are effective during treatment, they may not be as readily available or effective in the post-treatment phase. The review emphasises the importance of identifying and leveraging facilitators that resonate with BCS in their post-treatment lifestyle. This finding is consistent with the work of Puklin et al. [20], who noted the significance of healthcare provider support in promoting PA during treatment.

Moreover, the qualitative insights from BCS provide a deeper understanding of their lived experiences, motivations and preferences regarding PA. These insights are invaluable for designing patient-centred interventions that address the specific needs and concerns of BCS. By synthesising qualitative data, this review uncovers nuanced factors that quantitative studies may overlook, leading to more effective and personalised survivorship care plans (SCPs). This approach builds on the recommendations of Lavallée et al. [19], who emphasised the importance of understanding the barriers and facilitators to PA during treatment.

The review also bridges the gap between existing quantitative findings and the real-world experiences of BCS. By focusing on the post-treatment phase, this review contributes to the broader goal of improving long-term health outcomes and quality of life for BCS. The findings from this review can inform the development of targeted, effective and sustainable interventions that support the health and well-being of BCS.

In conclusion, this qualitative SR provides a comprehensive understanding of the barriers and facilitators to PA among BCS post-treatment. The findings highlight the need for tailored interventions that address the unique challenges and enablers faced by BCS in the post-treatment phase. By incorporating qualitative insights from BCS, this review contributes to the development of patient-centred interventions that enhance survivorship care and improve long-term health outcomes for BCS.

### 4.2. Strengths and Limitations

One of the key strengths of this study is the use of second reviewers during both the screening process—covering 20% of de-duplicated articles—and the quality appraisal stage. This methodological approach helps to reduce bias and increases the reliability of the conclusions drawn. Another notable strength is the diversity of the sample. Although the sample size was relatively small (*n* = 200), it included BCS from seven different countries, representing a range of ethnicities, races and cultures. This international scope provides valuable insight into cross-cultural experiences of BCS, enhancing the study's relevance despite the limited sample size.

During the data extraction process, it was noted that several articles lacked detailed participant characteristics (sociodemographic data), as well as diagnosis, treatment and survival details. This limitation hinders the ability to draw in-depth conclusions when comparing ethnicity, age and treatment type. It is recommended that future research on BCS consistently includes sociodemographic characteristics to identify commonalities within specific groups and enhance the specificity of support and care.

Five of the reviewed articles utilised voluntary PA interventions, indicating a willingness among these BCS to engage in PA. However, other BCS may be less inclined to participate, suggesting that survivors with little or no interest in PA are a hard-to-reach population for qualitative studies on PA experiences. Future research should consider incentivisation to increase participation and capture a diverse range of PA experiences, both positive and negative.

The authors also acknowledge the overrepresentation of positive results in the PA field [40]. To address this, all papers meeting the inclusion criteria were included, regardless of study findings. A comprehensive search strategy was employed to ensure the inclusion of unpublished studies and grey literature.

## 5. Conclusion

This research provides a strong rationale for incorporating PA as a structured component of SCPs. Personalised PA recommendations that align with patient preferences, offering flexible, community-based, or digital PA support programs to enhance long-term adherence, should be embedded within SCPs. The research also highlights that many BCS face barriers that negatively impact their PA participation and daily life. The findings from this review suggest that there is still a need for progress in the provision and support of PA for individuals undergoing or recovering from BC. While the results align with those of previous qualitative reviews, they reinforce a persistent gap: the continued lack of tailored PA interventions designed specifically for women with BC or BCS remains a significant issue. This should be systematically addressed through a multidisciplinary approach in survivorship care models (SCMs) to alleviate the hardships faced by BCS post-diagnosis. Healthcare providers, including oncologists, physiotherapists, psychologists and social workers, can collaborate to provide tailored interventions aimed at reducing these barriers. The findings of this review may serve as a foundation for future research and the development of SCMs.

## 6. Recommendations

Future research should focus on PA experiences from country-specific populations of BCS, utilising large and diverse samples reflective of national populations. This approach may result in a more accurate representation of findings, considering variations in incidence and survival rates, as well as differences in culture, healthcare systems and PA experiences across different countries. This could facilitate the creation and implementation of specific national PA guidelines for BCS in post-diagnosis life. A standardised global approach would be more specific and beneficial to all. Therefore, this research highlights the need for PA guidelines for BCS that consider cultural, healthcare and demographic variations. These guidelines can be incorporated into national survivorship care frameworks, ensuring that PA recommendations are both practical and widely applicable.

A semistructured approach is recommended to support those facing PA barriers while encouraging self-sufficiency. This structure would increase the flexibility desired by BCS [29, 32, 35]. Appropriate accessibility, facilities and funding are necessary for the successful continuation of PA.

## Figures and Tables

**Table 1:** SPIDER framework defined.

SPIDER	Review focuses
Sample^∗^	Female adult BCS population
Phenomenon of Interest	Experiences of PA
Design	Qualitative data collected through methods including focus groups and interviews
Evaluation	BCS' experience of PA in their postdiagnosis life
Research type	Qualitative studies (or mixed-method studies if contain separate qualitative results)

*Note:* This describes the SPIDER framework acronym used in qualitative systematic reviews [24] and the corresponding terms specific to this journal article for establishing the study's aim.

Abbreviations: BCS = breast cancer survivors, PA = physical activity.

^∗^Mixed-gender studies were included if they contained separate results between male and female. The female data could then be extracted without compromising the sample aim.

**Table 2:** Specific search strategy proximity technique terms.

Databases	Specific proximity search terms used	All other search terms used
APA PsycInfo	‘Breast cancer' N3 surviv^∗^	Experienc^∗^ AND ‘physical activity' OR ‘PA' OR sport^∗^ OR fitness OR exercis^∗^
CINAHL Plus		
SPORTDiscus		
OVID medline	‘Breast cancer' adj3 surviv^∗^	
Scopus	‘Breast cancer' W/3 surviv^∗^	
Sports medicine and education index	‘Breast cancer' NEAR/3 surviv^∗^	

*Note:* This table demonstrates the databases' differing methods of proximity searching with respect to the conjunction term between the wanted search terms, specific to identifying the two terms within three words.

^∗^Symbolises the truncation of the term; possibilities include survival, survivor, survivorship, etc.

**Table 3:** Eligibility criteria for search strategy screening.

Inclusion criteria	Exclusion criteria
Studies with sample of female adults (age 18+) who have previously had a diagnosis of breast cancer (any stage)	Studies involving anyone under age 18
Studies that discuss BCS' experience of PA or sport	Studies involving males in the sample (unless data presented separately for gender)
Studies that include BCS in any stage of cancer journey post diagnosis (treatment, remission, etc.)	Studies involving only experiences of anyone (carers/family) other than BCS' point of view^∗^
Studies from the BCS point of view only	Studies published not in English language
Studies with qualitative data including focus groups or interviews/semistructured interviews	Studies published out with the last 10 years (before January 2014)

*Note:* This table displays the criteria created by author NB used in the screening process of database hits, determining eligibility specific to the study's aims.

Abbreviations: BCS = breast cancer survivors and PA = physical activity.

^∗^Mixed-sample studies were included if they contained separate results between female BCS and other group. The BCS data could then be extracted without compromising the sample aim.

**Table 4:** Quality of final articles assessed using the CASP checklist [25].

CASP checklist	Articles									
	Balneaves et al. [29]	Chan et al. [30]	Hirschey et al. [31]	Inam et al. [32]	Kim et al. [33]	Milosevic et al. [34]	Owusu et al. [35]	Robinson et al. [36]	Sebri et al. [37]	Stalsberg et al. [38]
(1) Clear statement of aims?	Y	Y	Y	Y	Y	Y	Y	Y	Y	Y
(2) Qualitative methods appropriate?	Y	Y	Somewhat	Y	Y	Y	Y	Y	Y	Somewhat
(3) Design appropriate to address aims?	Y	Y	Y	Y	Y	Somewhat	Y	Somewhat	Can't tell	Somewhat
(4) Theoretical underpinnings clear, consistent and conceptually coherent?	Can't tell	Can't tell	N	Somewhat	Can't tell	Can't tell	Can't tell	Y	Somewhat	Can't tell
(5) Recruitment strategy appropriate to aims?	Y	Y	Somewhat	Y	Y	Y	Y	Y	N	Y
(6) Data collection methods address research issue?	Y	Y	Y	Y	Y	Y	Y	Y	N	Somewhat
(7) Relationship between researcher and participants adequately considered?	Y	Can't tell	N	Y	Y	Somewhat	Y	Somewhat	Somewhat	N
(8) Ethical issues considered?	Y	Y	Y	Y	Y	Y	Y	Y	Y	Y
(9) Data analysis sufficiently rigorous?	Somewhat	Y	Y	Y	Y	Y	Y	Y	Somewhat	N
(10) Clear statement of findings?	Somewhat	Y	Somewhat	Y	Y	Somewhat	Y	Y	Y	Y
(11) How valuable is the research?	Answers to question 11 can be found in text									
Quality conclusion	Medium	Medium	Medium	High	High	Medium	High	Medium	Low	Low

*Note:* This demonstrates the answered domains of the CASP checklist per each article and highlights the concluding quality assessment discussed by the two reviewers Neeve Brown and Fiona Muirhead. Y = Yes, N = No.

Abbreviation: CASP = Critical Appraisal Skills Programme.

**Table 5:** Main findings identified from final articles' results.

Main themes	Sub-themes
Outcomes of PA participation	Importance of social connections
	Physical and psychological impacts
	Optimistic outlook towards life ahead
	Self-management aspect to helping self

Barriers surrounding PA	Negative expectations, beliefs and myths surrounding PA
	Capabilities decreased due to side-effects
	Clashing commitments

Postdiagnosis balancing act	New you versus old you
	Comparing self to other BCS
	Conflicting debate of freedom against strictness

Needs for future PA programmes	Variation and flexibility in activities
	Semistructured approach
	Standardisation of accurate advice

Next steps for BCS	Desire for continuation of PA
	Playing an active role in research

*Note:* This table provides an outline of main themes and subthemes concluded from the final 10 articles included in this study.

Abbreviations: BCS = breast cancer survivors and PA = physical activity.

**Table 6:** Study characteristics.

Author year location	Study aim(s)	Sample characteristics (number, gender, race/ethnicity, mean age (years))^∗^	Breast cancer diagnosis and postdiagnosis details∗	Intervention involved (if applicable)	Study design	Data collection methods	Data analysis approach	Key findings—main themes
(Balneaves et al., 2014) [29] Canada	Experiences and perspectives of breast cancer survivors who had participated in a lifestyle intervention	*N* = 9 Female Caucasian 55.6	Stage II *n* = 6 Stage III *n* = 1 Stage unknown *n* = 2 Time since completion of treatment = 2.5 years	24-week pilot lifestyle intervention—PA and dietary	Qualitative	Focus group and semistructured telephone interviews	Thematic analysis	(1) Perceptions of the intervention (2) Perceived outcomes of the intervention (3) Perceived challenges of the intervention (4) Perceived facilitators of the intervention (5) Recommended changes to the intervention
(Chan et al., 2023) [30] China	Breast cancer survivors' perspectives on regular walking exercise to improve postchemotherapy neurotoxicity impairments	*N* = 15 Female Chinese Age range = 39.0–68.0	Stage I *n* = 1 Stage II *n* = 9 Stage III *n* = 5 All completed 4–8 rounds of chemotherapy	N/A	Qualitative	Semi-structured interviews	Manifest and latent content analysis	(1) The perceived effects of regular walking on neurotoxicity impairments (2) Unmet information needs (3) The regular-walking habit being self-sustained (4) Enablers and constraints of regular walking exercise
(Hirschey et al., 2017) [31] USA	Common exercise outcome expectations among 20 female survivors of Stage IA–IIB breast cancer who completed adjuvant treatment and an exercise intervention	*N* = 20 Female Caucasian (*n* = 15), African American (*n* = 5) 62.0	Time since completion of treatment = 4.2 years	RCT 16-week lab-based aerobic exercise intervention (within a year previous)	Mixed-methods	Semistructured telephone interviews and modified outcome expectations for exercise questionnaire	Summative content analysis and latent analysis	(1) Prevalence of common expectations (2) Pervasive impact of fatigue (3) A brighter future
(Inam et al., 2023) [32] Australia	Components of PA programmes considered important by women who had breast cancer as well as the allied health staff that support them	*N* = 18 (BCS *n* = 11 and allied health professionals *n* = 7) Female BCS	All participants completed their primary treatment at 0.5 years prior	N/A	Qualitative	Focus group and semi-structured interviews	Thematic analysis	(1) The need for PA programs (2) Person-centred programmes (3) Flexible PA programmes (4) Systems factors
(Kim et al., 2019) [33] Korea	Experience of cancer-related fatigue among BCS, barriers to exercise and perceived facilitative factors to overcome fatigue and identify implications for future exercise adherence programmes	*N* = 16 Female Korean 48.0	Stage I *n* = 6 Stage II *n* = 10 Time since diagnosis = < 1 year All but *n* = 4 BCS completed treatment	N/A	Qualitative	Focus groups	Thematic analysis	(1) The insidious and overpowering nature of cancer-related fatigue (2) Exercising when experiencing fatigue surrounded by prevailing myths (3) Multiple barriers to exercise (4) Facilitative factors to continue exercising despite fatigue (5) Suggestions for clinical practice and exercise adherence programmes
(Milosevic et al., 2020) [34] Canada	Young breast cancer survivors' beliefs and practices regarding PA, healthy eating and weight management	*N* = 12^∗∗^ Female Asian (*n* = 2), White (*n* = 8), Black (*n* = 1) 36.0	All participants treated with chemotherapy or combination chemotherapy	N/A	Qualitative	Semistructured telephone interviews	Thematic analysis	(1) Prolonging life with a healthy lifestyle versus enjoying living (2) Perceiving benefits versus barriers (3) Seeking social connection versus protecting the self from social threats
(Owusu et al., 2018) [35] USA	Beliefs, attitudes and preferences of older BCS from diverse racial and socioeconomic backgrounds towards PA to inform the design of a PA program that fosters acceptability	*N* = 60 Female Non-Hispanic White (*n* = 30) African-American (*n* = 30) Age range = 65.0–87.0	Stage I *n* = 32 Stage II *n* = 20 Stage III *n* = 8 Time since treatment completed = < 2.0 years (surgery, chemotherapy and/or radiation therapy)	N/A	Qualitative	‘One-on-one' interviews and follow-up focus groups	Thematic analysis	(1) Importance of PA (2) Current PA participants engaged in (3) Influence of race and culture on PA attitudes and beliefs (4) Barriers and facilitators (5) Preferences for a PA programme
(Robinson et al., 2015) [36] USA	Examine breast cancer survivors' motivation for participating in team triathlon training, completing a triathlon and sustained exercise thereafter	*N* = 11 Female 50.9	All participants Stage 0-III Average length of survivorship = 5.0 years	14-week individualised team sprint triathlon training programme	Qualitative	Focus groups and individual telephone interviews	Content analysis	(1) Champion for exercise (2) Part of a team (3) everyone had a story (4) Not really exercise (5) What do we do now?
(Sebri et al., 2022) [37] Italy	Initial motivations and perceived outcomes in female cancer survivors' participation in two different programs that combine sports activities and group psychological support to promote quality of life	*N* = 45 Female 50.1	All participants in the post-treatment stage	PA (postural exercises and sailing boat courses) and psychological group intervention—21 sessions in total	Qualitative	Open-ended questionnaire	Thematic analysis	(1) Physical well-being (2) Psychological well-being (3) Coping with illness (4) Social relationships (5) Support to research
(Stalsberg et al., 2019) [38] Norway	Identify levels of daily routines for, and experiences with PA among long-term BCS, in general and on the part of SES groups	*N* = 37 (interviews) Female 61.0	N/A	Longitudinal follow-up study concerning health-related quality of life and late effects	Mixed-methods	Semistructured interviews and activity-logs	Parallel mixed analysis	(1) Positive associations to PA (2) Fulfilling ambitions or not (3) PA constraints (4) The art of balancing duties and leisure time activities (5) To appear physically active (6) Strategies for PA

*Note:* This table demonstrates the data extraction stage summarised into key characteristics of the final 10 articles.

Abbreviations: BCS = breast cancer survivors, PA = physical activity.

^∗^All available data reported within each study were documented.

^∗∗^ *n* = 1 participant did not wish to report any sociodemographic data to the original article.

## Data Availability

The data that support the findings of this study are available from the corresponding author upon reasonable request.

## Appendix A

Data extraction results outlined by studies' characteristics (Table A1).

## Supporting Information

The supporting file includes the modified version of the CASP tool [25], which the authors used for this review. The modified version includes an additional question and a fourth response option, ‘somewhat', indicating a reasonable attempt at fulfilling a quality domain with clear strengths and limitations. The assessment involved scoring 11 questions regarding methodological quality as ‘yes', ‘can't tell', ‘no' and ‘somewhat'. These questions addressed research design, recruitment strategy, data collection, ethics, data analysis and presentation and interpretation of findings.
